# Sex and Immunogen-Specific Benefits of Immunotherapy Targeting Islet Amyloid Polypeptide in Transgenic and Wild-Type Mice

**DOI:** 10.3389/fendo.2016.00062

**Published:** 2016-06-14

**Authors:** Pavan K. Krishnamurthy, Hameetha B. Rajamohamedsait, Veronica Gonzalez, Wajitha J. Rajamohamedsait, Nawal Ahmed, Senthilkumar Krishnaswamy, Einar M. Sigurdsson

**Affiliations:** ^1^Department of Neuroscience and Physiology, NYU School of Medicine, New York, NY, USA; ^2^Department of Psychiatry, NYU School of Medicine, New York, NY, USA

**Keywords:** diabetes, glucose, islet amyloid polypeptide, immunotherapy, insulin, pancreas, survival curves

## Abstract

Type 2 diabetes mellitus is characterized by the deposition of islet amyloid polypeptide (IAPP) as amyloid in islets, a process thought to be toxic to β-cells. To determine the feasibility of targeting these aggregates therapeutically, we vaccinated transgenic (Tg) mice that overexpress human IAPP and were fed a high-fat diet to promote their diabetic phenotype. Our findings indicate that prophylactic vaccination with IAPP and its derivative IAPP_7-19-TT_, protects wild-type female mice, but not males, from obesity-induced early mortality, and the derivative showed a strong trend for prolonging the lifespan of Tg females but not males. Furthermore, IAPP_7-19-TT_-immunized Tg females cleared a glucose bolus more efficiently than controls, while IAPP-immunized Tg females showed an impaired ability to clear a glucose bolus compared to their adjuvant injected Tg controls. Interestingly, IAPP or IAPP_7-19-TT_ treatments had no effect on glucose clearance in Tg males. Overall, these beneficial effects of IAPP targeted immunization depend on Tg status, sex, and immunogen. Hence, future studies in this field should carefully consider these variables that clearly affect the therapeutic outcome. In conclusion, IAPP targeting immunotherapy may have benefits in patients with type 2 diabetes.

## Introduction

Insulin, produced by β-cells in the pancreas, has the key function of regulating blood glucose levels after food intake. In the early stages of type 2 diabetes, insulin production is normal or increased in absolute terms, but this is insufficient to control blood glucose levels due to reduced insulin sensitivity (1). Additionally, there may also be a deficit in the ability of the pancreas to secrete insulin to closely match the temporal rise in blood glucose levels, for example, immediately following a meal, resulting in hyperglycemia.

Islet amyloid polypeptide (IAPP or amylin) is also secreted by the β-cells (2). Its proposed functions include to inhibit insulin secretion (3) and glucagon secretion (4), although contradictory findings have also been reported (5). IAPP can also aggregate and form amyloid deposits in the pancreas and, thus, is considered a pathological hallmark of type 2 diabetes (6). Post mortem studies indicate that between 40 and 90% of patients with type 2 diabetes show signs of amyloidosis, and amyloid formation is a significant factor in the deterioration of islet function and reduction in β-cell mass [reviewed in (7)]. Interestingly, the process of IAPP deposition in the pancreas has been likened to amyloid-β (Aβ) deposition in the brain of Alzheimer’s disease patients.

With an increasing understanding of the complicated pathophysiology seen in type 2 diabetes, treatment strategies aimed at achieving glycemic control need to be updated. A combination of dietary changes, exercise and prophylactic treatments to improve insulin sensitivity, preserve β-cell function, and promote weight loss has been articulated (8). While dietary changes and physical activity may reduce symptomatic burden, these measures generally do not reverse the pathogenesis of the disease. Neither do available drugs that provide merely symptomatic relief. As IAPP aggregates are a prominent feature of type 2 diabetes that may be involved in its pathogenesis, targeting these for clearance is an attractive approach. This can potentially be accomplished with immunotherapy. Currently, the potential of using a vaccine to remove protein aggregates found in several neurodegenerative diseases is undergoing extensive research in *in vivo* models of disease as well as in clinical trials. For example, immunotherapy targeting Aβ and tau deposits in the brain of Alzheimer’s disease patients, as well as α-synuclein deposits seen in Lewy Body disease or Parkinson’s disease, and against misfolded copper/zinc superoxide dismutase 1 protein in a mouse model of amyotrophic lateral sclerosis are at various stages of development [reviewed in (9)]. Thus, the idea of using immunotherapy to clear or prevent IAPP deposits seen in type 2 diabetes holds considerable promise as a therapeutic strategy.

In the current study, we investigated the feasibility of using an immunotherapeutic approach to target IAPP in a mouse model of type 2 diabetes. Our approach was to design an immunogen that contains the B-cell epitope of IAPP but is void of the hydrophobic region that confers fibrillogenicity and thereby toxicity under certain conditions. This is analogous to our approach targeting Aβ in Alzheimer’s disease (10–12). To promote antibody response toward such a shortened sequence, tetanus toxin fragment (TT947-967), T-helper epitope that is commonly used in various marketed vaccines was attached to the B-cell epitope of IAPP. We used an IAPP derivative (αα7-19 linked to tetanus toxin) or unmodified IAPP as vaccines to prophylactically (2–14 months) treat transgenic (Tg) mice that express human IAPP (hIAPP) in their pancreatic β-cells (13). About two-thirds of male hIAPP mice, but only 10% of female hIAPP Tg mice, have been reported to develop islet amyloid after 12–16 months on a high-fat diet (14). To assess the diabetic phenotype of the mice, their weights and plasma glucose levels were monitored at regular intervals. At the study endpoint, their ability to clear a glucose bolus was assessed as well as plasma insulin levels, followed by histological analysis of pancreatic tissue to determine if the vaccines cleared IAPP deposits and had any effect on total β-cell area. Wild-type (WT) mice were included as controls.

Our findings indicate that prophylactic vaccination with IAPP or its derivative, IAPP_7-19-TT_, reduces substantially death in WT female mice, with the derivative showing a strong trend for lowering mortality in Tg females as well. The most significant benefits were observed when these two female groups were combined for analysis. In addition, sex and immunogen-specific effects were detected in Tg animals’ ability to clear the glucose bolus. Tg females immunized with the IAPP derivative had improved glucose clearance compared to controls. IAPP-immunized Tg females were impaired in their glucose clearance, which may be explained by their higher antibody-titer against IAPP than in the derivative group.

## Materials and Methods

### Mouse Model

Human islet amyloid polypeptide Tg mice were obtained from VA Puget Sound Health Care System/University of Washington, Seattle, WA, USA. These mice, which have been extensively characterized (13–16), were bred to obtain hemizygous hIAPP mice on a mixed DBA/2-C57Bl/6 background. F1 mice born from C57BL/6 Tg male mice bred with DBA/2 non-Tg females were used in this study. The genotypes of the mice were confirmed by PCR of tail DNA with appropriate sense and antisense primers as previously reported (17). Non-Tg WT littermates were also used. All animal procedures were approved by the Institutional Animal Care and Use Committee (IACUC) at the New York University School of Medicine and were in accordance with the National Institutes of Health’s Guide for the Care and Use of Laboratory Animals.

To promote the deposition of hIAPP-derived islet amyloid, mice were maintained on a high-fat diet [Diet 12,290 (45% kcal fat, 35% kcal carbohydrates, 20% kcal protein); Research Diets] beginning at 2 months of age. They were maintained on a 12 h light/dark cycle in an AAALAC approved pathogen-free facility at the New York University School of Medicine and monitored daily by veterinary staff and/or the investigators. The animals were humanely euthanized at the end of the experimental period at 14 months with sodium pentobarbital (100 mg/kg, intraperitoneal (i.p.) and perfused trans-aortically with phosphate buffered saline (PBS) containing heparin. At this age, the animal should have developed substantial IAPP deposits in the pancreas. Animals that appeared to be sick and suffering during the experimental period were euthanized with the same dose of sodium pentobarbital (5 out of 251). Standard criteria used for early termination includes weight loss, inappetence, infection not responding to treatment and accompanied by systemic signs of illness, and signs of severe organ system dysfunction with poor prognosis as determined by veterinarian.

### Vaccine Treatment

At 2 months of age, mice were bled and assigned for treatment with hIAPP derivative IAPP7-19-GPSL-Tetanus Toxin947-967 (IAPP_7-19-TT_), full length hIAPP (IAPP) or control. Both peptides were synthesized at the W. M. Keck Foundation, Yale University (New Haven, CT, USA) by solid-phase technique and purified by high-performance liquid chromatography. The vaccines were administered in alum adjuvant (Adju-Phos®, Brenntag; overnight mixing at 4°C), which promotes primarily antibody production and has a lesser chance of eliciting T-cell-related side effects (18). The mice received an i.p. injection of 100 μg/100 μl followed by a second injection 2 weeks later and then monthly thereafter until 14 months of age. Mice were killed when they were ~14–14.6 months old. Control mice received 100 μl alum adjuvant alone.

### Antibody Response

Mice were bled prior to their first treatment and subsequently after every third injection until the study endpoint at 14 months of age. The antibody response to IAPP_7-19-TT_ or IAPP was determined by a 1:200 dilution of plasma using ELISA, essentially as described previously (10, 11). The response was detected with goat anti-mouse IgG linked to horseradish peroxidase, 1:3000 (Thermo), with tetramethyl benzidine (TMB) as the substrate.

### Plasma IAPP Levels

Islet amyloid polypeptide levels were measured from a random selection of the final bleeds using the human IAPP ELISA kit (EMD Millipore). Plasma samples were diluted and assayed as per the manufacturer’s instructions.

### Intraperitoneal Glucose Tolerance Test

An i.p. glucose tolerance test (IPGTT) was carried out as described previously (15) with a few minor modifications. Mice were fasted for 16–18 h overnight and then anesthetized using sodium pentobarbital (50 mg/kg i.p.; Ovation Pharmaceuticals). The standard mouse anesthetic is not recommended as it affects glucose metabolism. After approximately 40 min, blood was drawn from the retro-orbital sinus to determine fasting plasma glucose levels. Thereafter, mice were injected i.p. with 10 μl/g body weight of 10% dextrose and blood was taken at 5, 15, 30, 60, 90, and 120 min following commencement of the glucose bolus. The blood samples were used to determine plasma glucose levels.

### Histology

One week following the IPGTT procedure, mice were again fasted overnight and sacrificed to obtain terminal plasma samples and tissue for analysis. Mice were anesthetized with sodium pentobarbital (100 mg/kg, i.p.) and perfused trans-aortically with PBS containing heparin. The pancreas was excised and split in half: one portion was frozen and stored at −80°C for biochemical assays and the other immersion-fixed in periodate-lysine-paraformaldehyde buffer (PLP) and subsequently embedded in paraffin. From the embedded portion, 5 μm sections were cut and sections at 100 μm intervals were first deparaffinized in xylene and then stained with: (1) 0.5% thioflavin S (filtered), (2) anti-IAPP 1:200 (Peninsula Labs, CA, USA) in polyclonal diluent (2% Triton-X, 0.1% sodium azide, 0.01% bacitracin, 2% bovine serum albumin, 10% normal serum in PBS pH 7.4), and (3) anti-insulin 1:1000 (Sigma) in monoclonal diluent (MOM kit, Vector Labs, CA, USA). Prior to IAPP and insulin staining, antigen retrieval was carried out by boiling the sections in 0.2% citrate buffer for 10 min (IAPP) or dipping them for the same amount of time into 40% formic acid (insulin). The immunostained sections were revealed with 0.3% hydrogen peroxide in 3,3-diaminobenzidine tetrahydrochloride with nickel ammonium sulfate intensification. Slides were re-hydrated and dehydrated and coverslipped for analysis. The selection of tissue for analyses was completely unbiased and based on a random sampling from the excised half portion of fixed pancreas. The prevalence and severity of amyloid in this model has been found to be uniform throughout the pancreas (19).

### Biochemical Assays

Blood samples were collected at various time points during the course of vaccine or control adjuvant treatment and also at the terminal endpoint 1 week following the IPGTT procedure. Mice were fasted overnight and sacrificed to obtain terminal blood samples. Plasma glucose was measured using a glucose meter and glucose strips (Accu-Chek Active®, Roche).

Plasma samples obtained from the IPGTT bleeds were also used to determine plasma insulin levels by ELISA using a commercially available kit [Insulin (Mouse) High and Low Range ELISA, ALPCO Diagnostics, NH]. Plasma glucose levels were measured from fed mice during the course of vaccine or adjuvant treatment, and from fasted mice during the IPGTT measurements.

### Image Analysis

Images were acquired with a Leica DM5000B microscope and MBF Bioscience software. Images were then merged with Photoshop (Adobe) to obtain a composite image of the entire tissue section. The area of pancreas (islet) staining was quantified with NIH Image J software. The percentage of the total area stained for IAPP or insulin was calculated as total IAPP or insulin positive area (square micrometer), i.e., area occupied by the reaction product, divided by the total section area (square micrometer) and expressed as percentages. Amyloid deposits were detected with Thioflavin S and the percentage of positive islet area calculated. Islets that were not Thioflavin S positive could be easily identified on these sections.

### Statistical Analysis

Data analysis was done with Graph Pad Prism 5 (GraphPad Software, Inc., La Jolla, CA) and StatSoft Statistica 6 (StatSoft, Inc., Tulsa, OK) software. Mice were analyzed based on treatment, sex, and genotype, for example, control female Tg mice versus IAPP_7-19-TT_-treated female Tg mice. Kaplan–Meier survival graphs were plotted for all mice till the time of IPGTT. Survival curves were compared using Mantel–Cox (log-rank) test and median survival was only reported when survival was below 50%. Plasma glucose, fasting plasma glucose, and IPGTT plasma glucose levels were analyzed by two-way ANOVA, repeated measures and Newman–Keuls *post hoc* test. Plasma insulin and histological analysis of insulin, IAPP, and Thioflavin S immunoreactivity of vaccinated versus control adjuvant treated groups was analyzed by one-way ANOVA. When the data failed at least two of three normality tests (Kolmogorov–Smirnov, D’Agostino and Pearson omnibus, and Shapiro–Wilk normality tests), non-parametric Kruskal–Wallis test was used. The test was two-tailed as the levels could be expected to be increased or decreased. Data are expressed as mean ± SEM. A *p* ≤ 0.05 was considered significant.

## Results

### Survival – IAPP_7-19-TT_ Study

**Figure 1:**
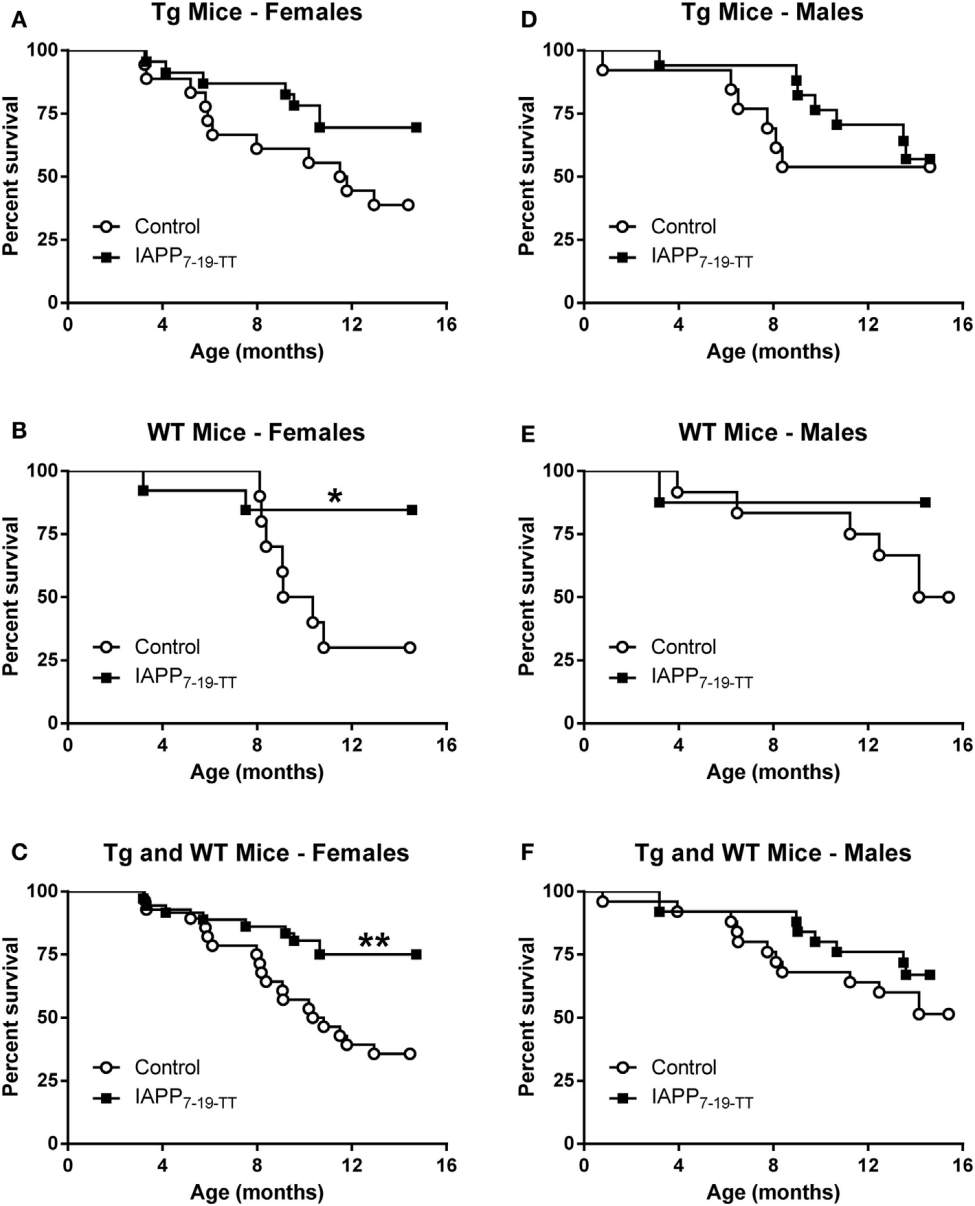
**Prophylactic IAPP_7-19-TT_ vaccination promotes survival in Tg and WT female mice**. Kaplan–Meier survival plots for mice enrolled in the prophylactic IAPP_7-19-TT_ study. **(A–C)** Tg and WT female mice receiving IAPP_7-19-TT_ treatment had a better survival outcome than their control adjuvant treated counterparts (*p* = 0.056 (Mantel–Cox test) for Tg mice, *p* = 0.02 for WT mice, and *p* = 0.002 for combined Tg and WT mice). **(D–F)** Prophylactic IAPP_7-19-TT_ treatment did not significantly improve survival of Tg or WT male mice.

Table 1 shows the numbers of mice of different sex and genetic background that were enrolled and died in the prophylactic IAPP_7-19-TT_ experiment starting at 2–3 months of age. Mice received monthly injections of IAPP_7-19-TT_, a derivative of IAPP in alum adjuvant or adjuvant alone. A number of mice did not survive until the study endpoint and died during the 12-month course of the study (Table 1). As both WT and Tg mice died prematurely, it was unrelated to overexpression of hIAPP but was likely related to their obesity. Kaplan–Meier analyses of Tg and WT littermate mouse deaths during prophylactic IAPP_7-19-TT_ or control treatment was carried out. Analysis of the survival curves indicated that the IAPP_7-19-TT_-treated Tg female mice trended strongly to survive longer than control (adjuvant alone) Tg female mice (*p* = 0.056), with a hazard ratio of 2.5, i.e., two and a half times less likely to die than controls (Figure 1A). Significantly more IAPP_7-19-TT_-treated WT female mice survived till the study endpoint than control treated mice (84.6% versus 30%, *p* = 0.02), and had a fivefold less chance of dying, hazard ratio = 5.11 (Figure 1B). Combining Tg and WT female mice showed that a significantly higher proportion of the IAPP_7-19-TT_-treated mice survived till the end of the study than control treated mice (75% versus 35.7%, *p* = 0.002) with a hazard ratio of 3.4 (Figure 1C). Considering these robust differences in survival, it is important to note that the surviving animals may not accurately represent the original group.

**Table 1:** **Mice enrolled and mouse deaths during the prophylactic IAPP_7-19-TT_ study**.

Treatment	Enrolled	Female Tg	Male Tg	Female WT	Male WT	End of study euthanized	Female Tg	Male Tg	Female WT	Male WT	Deaths	
											Found dead prior to IPGTT	During/post IPGTT
Control	53	18	13	10	12	20	5	6	3	6	29	4
IAPP_7-19-TT_	61	23	17	13	8	36	12	9	9	6	17	8

There were no significant differences in the survival proportions of IAPP_7-19-TT_ or control-treated Tg or WT male mice (Figures 1D–F) although both treated groups showed a trend for improved survival compared to their controls.

Further analysis of the weight data showed a similar slope of weight gain in mice that died during the study compared to mice that survived until the end of the study (data not shown), suggesting that increased mortality could not be explained by abnormal weight gain and related complications or by weight loss because of disease in the colony.

### Weight – IAPP_7-19-TT_ Study

To induce a diabetic phenotype, all the mice enrolled in the study were fed a high-fat diet from 2 months of age till the terminal end point. There were no significant differences in the weights of the groups (Figure S1 in Supplementary Material).

### Antibody Response and Plasma IAPP Levels – IAPP_7-19-TT_ Study

**Figure 2:**
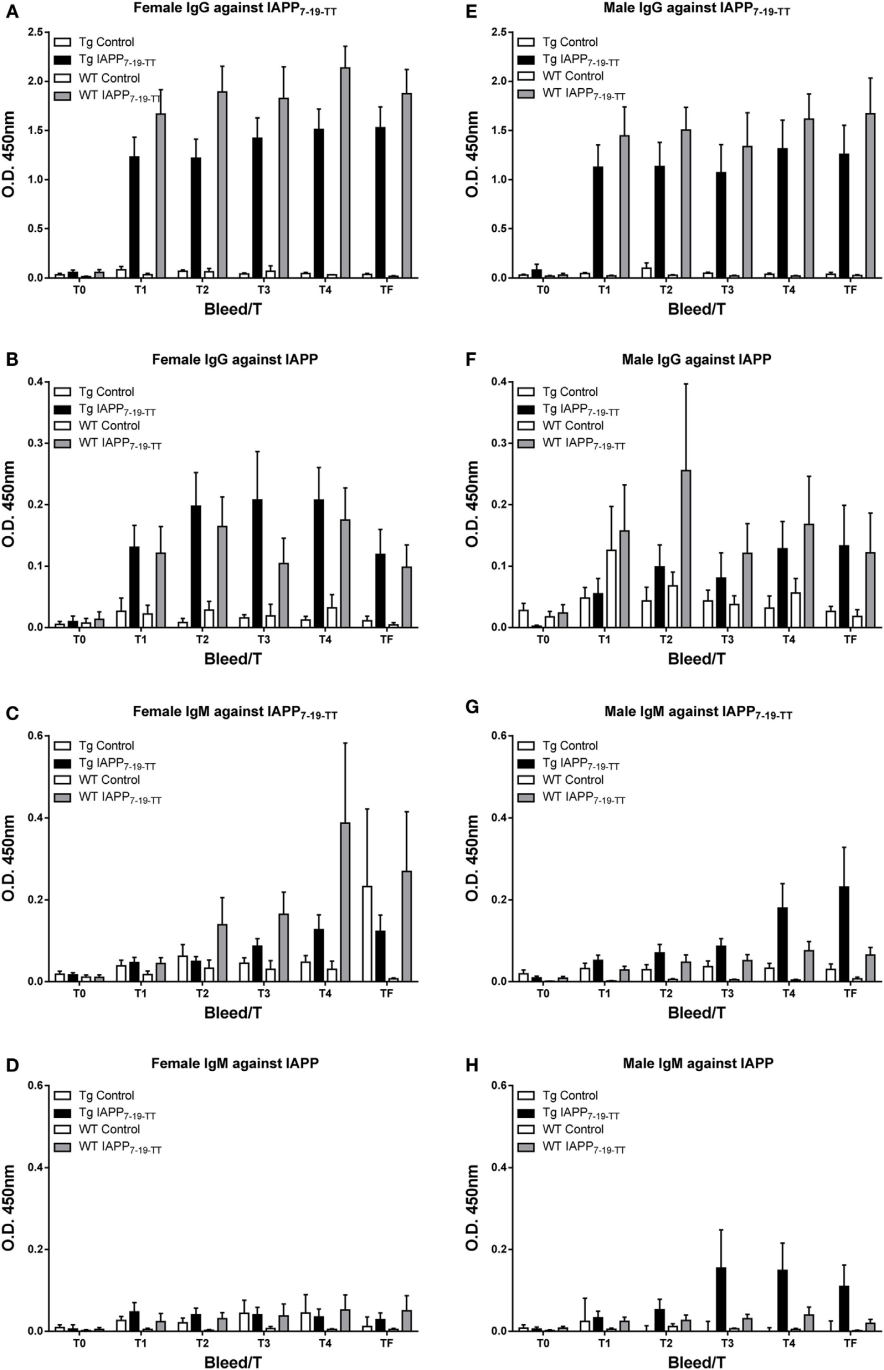
**IAPP_7-19-TT_ is highly immunogenic in mice but elicits a modest antibody response toward IAPP**. Tg and WT mice were immunized from 2 months of age with alum adjuvant (Control) or IAPP_7-19-TT_ peptide in alum adjuvant (IAPP_7-19-TT_). Mice were bled at regular intervals (T0–TF) and plasma samples (1:200) from the animals were analyzed by ELISA. IAPP_7-19-TT_-treated mice developed a strong and sustained IgG immune response to the immunogen **(A,E)** and a more modest response to IAPP **(B,F)**. IgG levels **(A,B,E,F)** were higher than IgM levels **(C,D,G,H)**. Error bars indicate SEM.

The antibody response toward the IAPP_7-19-TT_ immunogen was robust and comparable between sexes and the various treated groups (Tg and WT). IgG levels were higher than IgM levels. As expected, these antibodies showed a more modest reactivity toward IAPP (Figure 2).

Random sampling of Tfinal plasma samples from the study revealed increased plasma IAPP levels in immunized mice (WT: 466 (average) ± 109 (SEM) pmol/l, *n* = 13; Tg: 2093 ± 429, *n* = 19) compared to control mice (WT: 22 ± 5, *n* = 7; Tg: 149 ± 104, *n* = 9), indicating target engagement of the antibodies generated toward the vaccine. Sex differences were not apparent and plasma IAPP levels from Tg mice that had died during the course of the study (T3–T4) also had a comparable increase (Tg immunized: 950 ± 421, *n* = 6; Tg control: 134 ± 3, *n* = 7).

### Glucose Levels – IAPP_7-19-TT_ Study

Measurements of plasma glucose levels during the course of treatment (12 months) showed a significant decrease in plasma glucose levels prior to terminal sacrifice (T0–T4) for Tg female (*p* = 0.003), and male mice (*p* = 0.004; Figures S2A,C in Supplementary Material). However, glucose levels did not differ between control and treated Tg groups and there was not an interaction between the two factors (bleeds and treatment). Plasma glucose also decreased significantly over the course of the five bleeds (T0–T4) within WT female (*p* = 0.001) and WT male mice (*p* < 0.0001; Figures S2B,D in Supplementary Material). Again, there were no differences between the treatment groups or an interaction between the two factors (treatment and bleeds).

As the glucose tolerance test was performed between bleeds T4 and Tfinal, it may potentially influence Tfinal levels. Hence, terminal bleed plasma glucose levels were analyzed separately from T0 to T4. No treatment effect was observed in plasma glucose levels from the terminal bleed (Figure S2E in Supplementary Material).

Based on these measurements, the mice are not developing a severe diabetic phenotype, in spite of being fed a high-fat diet and gaining weight during the course of the study. Hence, it may be difficult to assess treatment effect based on this parameter. The glucose tolerance test is more appropriate to detect subtle differences in diabetic phenotype.

### Glucose Tolerance Test – IAPP_7-19-TT_ Study

**Figure 3:**
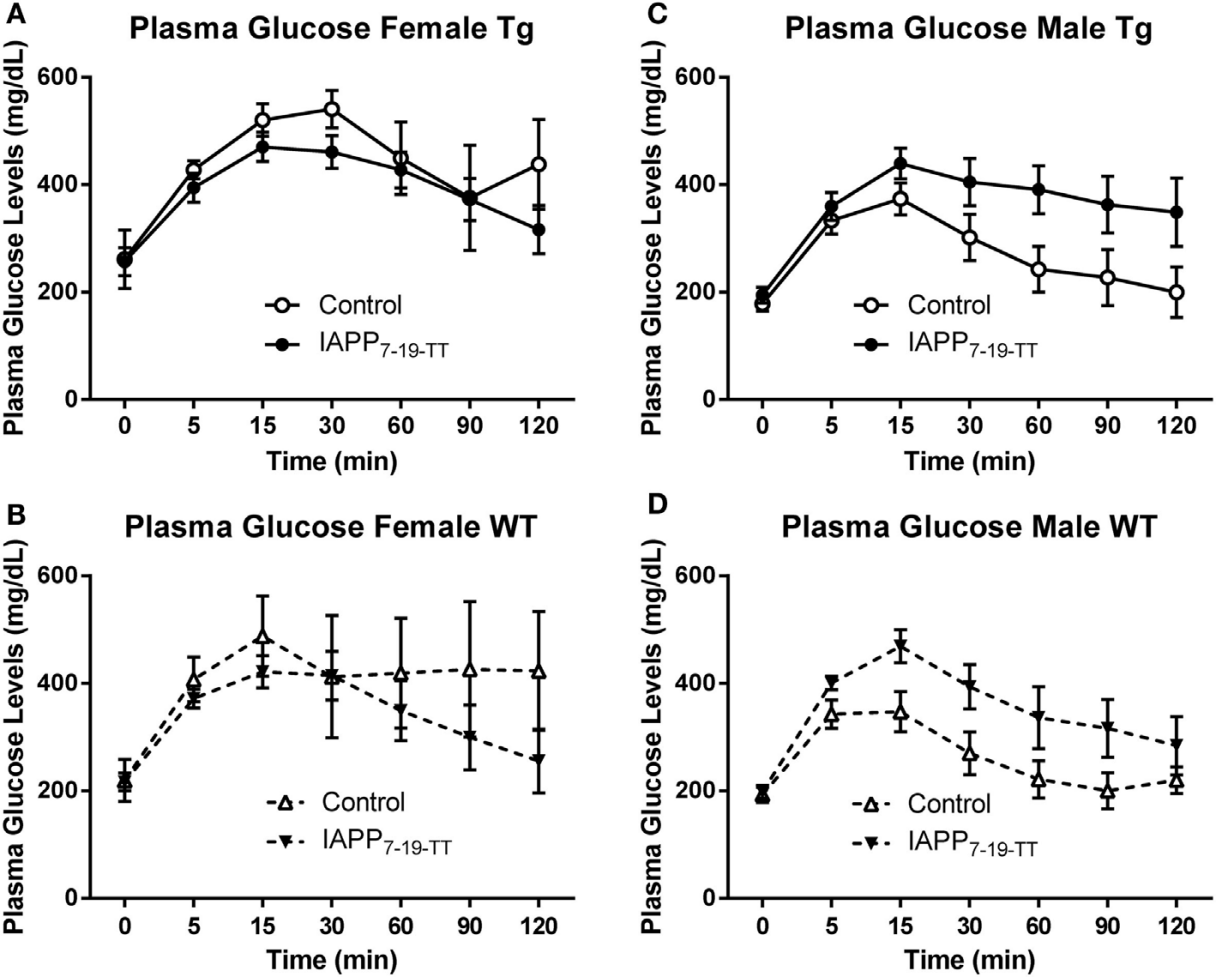
**Prophylactic IAPP_7-19-TT_ treatment attenuates glucose clearance in female Tg and WT mice**. At approximately 14 months of age, the diabetic status of the mice was assessed by the intraperitoneal glucose tolerance test (IPGTT). Mice were starved overnight and then bled to determine fasting plasma glucose levels (T0). They received a glucose bolus and were bled at various time points until T120 min. **(A,B)** IAPP_7-19-TT_-treated female Tg and WT mice trended to be able to clear the glucose bolus faster than control mice. **(C,D)** IAPP_7-19-TT_-treated male Tg or WT mice trended to clear the glucose bolus less efficiently than their controls. Error bars indicate SEM. Note that the increase in average values in the control group at 120 min in **(A)** is because one mouse, with a substantial drop in glucose levels at 90 min, died before the 120-min time point. A biphasic response was not seen in any of mice that went through this test, indicating that the procedure was performed correctly. Such errors can occur when the glucose is injected into the intestines instead of entering the peritoneal cavity.

To assess their diabetic status, 1 week prior to the end of the study period, mice were fasted for 16 h and then underwent an IPGTT. The test clearly worked as it was supposed to as evident by highly significant differences in glucose levels with time (*p* < 0.0001) in all groups analyzed (Figures 3A–D). Data were also analyzed as area under the curve (AUC) but it did not provide additional insight.

#### Females

The IAPP_7-19-TT_ treated Tg mice appeared to clear the glucose bolus more efficiently than the Tg controls, as was anticipated. Although a significant treatment effect was not observed in the Tg mice (*p* = 0.15), a significant interaction between time and treatment was detected (*p* = 0.002, Figure 3A). These findings indicate that clearance of the glucose bolus over time differed between the Tg treated and control mice. The WT IAPP_7-19-TT_ treated and controls did not differ significantly in glucose clearance, although plasma glucose levels remained elevated for the control-treated mice and did not return to basal levels as for the IAPP_7-19-TT_-treated mice (Figure 3B). Both Tg and WT control mice trended to being less efficient at clearing the glucose bolus than their IAPP_7-19-TT_-treated counterparts (Figures 3A,B), indicating that IAPP_7-19-TT_ treatment was attenuating the diabetic phenotype.

#### Males

There was a trend for the IAPP_7-19-TT_-treated Tg (treatment effect: *p* = 0.08) to clear the glucose bolus less efficiently than their controls, but not for WT (*p* = 0.11). This was also reflected within the Tg animals in differences in glucose clearance over time between the treatment groups (treatment × time interaction: *p* = 0.05; Figures 3C,D). IAPP_7-19-TT_-treated Tg mice showed a trend (*p* = 0.08), and IAPP_7-19-TT_-treated WT mice had significantly higher plasma glucose levels (*p* = 0.05) compared to control-treated mice (data not shown). These results were contrary to what we had anticipated if the treatment was attenuating a diabetic phenotype. The Tg controls did not show any significant differences in glucose levels when compared to their WT counterparts, which suggests a lack of diabetic phenotype in these male Tg mice. Hence, these effects may reflect immunization-mediated clearance of IAPP and, therefore, lessened ability to regulate spikes in glucose levels.

### Insulin Levels – IAPP_7-19-TT_ Study

Plasma insulin levels from the terminal bleeds were not significantly changed in the Tg or WT controls versus IAPP_7-19-TT_-immunized mice. There were no significant differences either between these groups when separated by sex (Figure S3 in Supplementary Material).

### Histology – IAPP_7-19-TT_ Study

**Figure 4:**
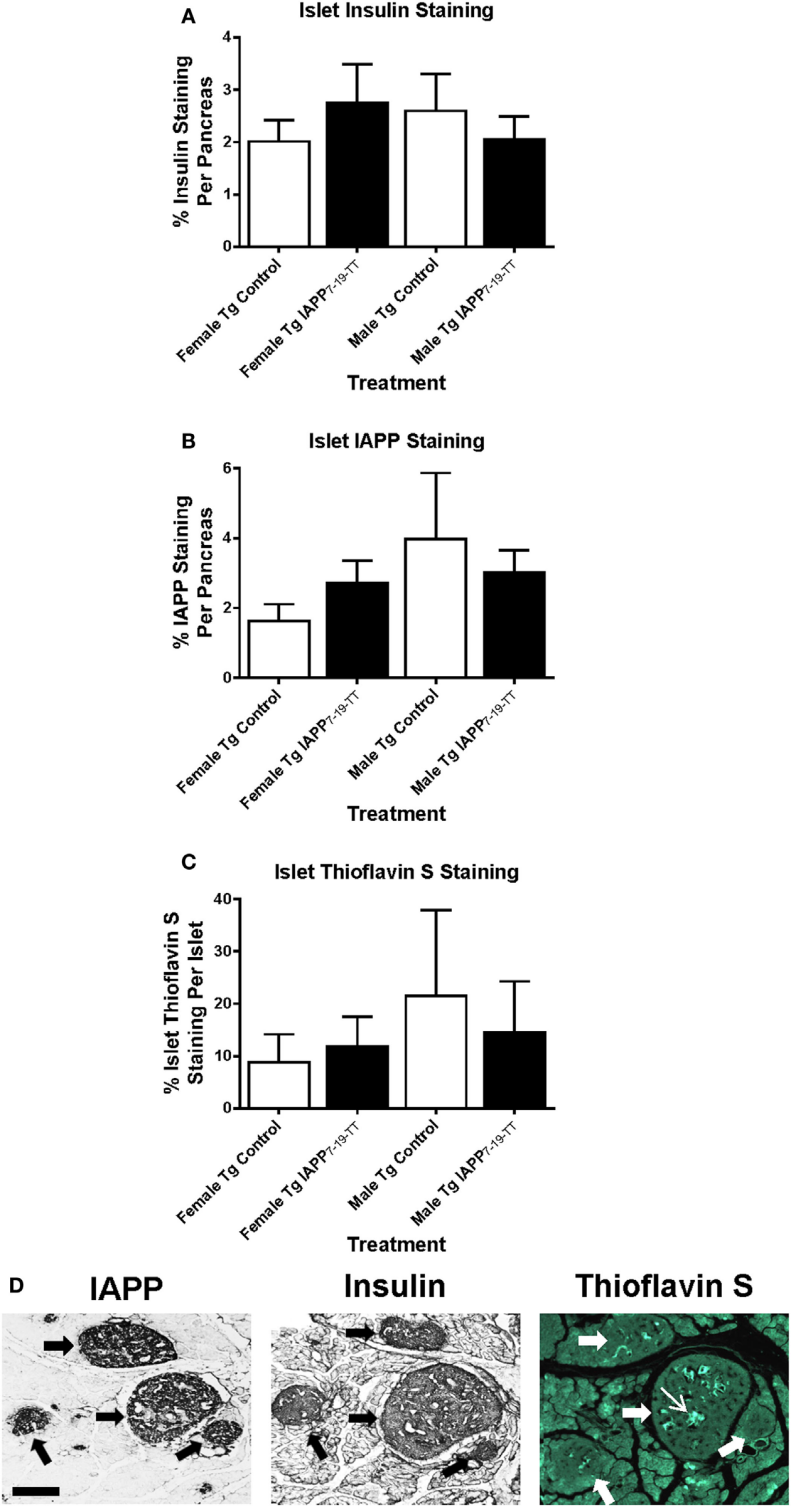
**Relative pancreatic β-cell area or amyloid burden are not changed in IAPP_7-19-TT_-immunized Tg mice versus controls**. Pancreatic tissue was collected at the study endpoint and used for histological analysis of insulin, IAPP, and amyloid load as determined by thioflavin S staining. **(A–C)** Relative islet β-cell area and amyloid burden were not altered in treated mice versus controls. Error bars indicate SEM. **(D)** Representative immunostaining of pancreatic tissue from a Tg mouse with some Thioflavin S positive IAPP deposits in the islets. Pancreatic tissue was sectioned and immunostained with anti-IAPP and insulin antibodies and processed for thioflavin S staining. Large arrows point at four islets of different sizes. The narrow arrow points at Thioflavin S positive deposit within one of the islets. Scale bar = 250 μm.

To obtain information about relative β-cell area in these animals, insulin and IAPP immunostaining was performed on Tg female and male mice (Figures 4A,B). The treatment groups did not differ within each sex.

Thioflavin S staining indicated that amyloid burden was very variable between controls and IAPP_7-19-TT_-immunized mice, and there were no significant differences between these groups for either sex (Figure 4C). Representative images show serial pancreatic tissue sections from an IAPP_7-19-TT_-treated Tg mouse with islet IAPP and insulin staining as well as some amyloid deposits stained with Thioflavin S (Figure 4D).

Pancreas sections from WT female and male-treated and control mice were also immunostained for insulin immunoreactivity to assess relative β-cell area in these animals. Analysis of the sections indicated no significant differences in β-cell area staining between control and treated groups within each sex (data not shown).

### Survival – IAPP Study

**Figure 5:**
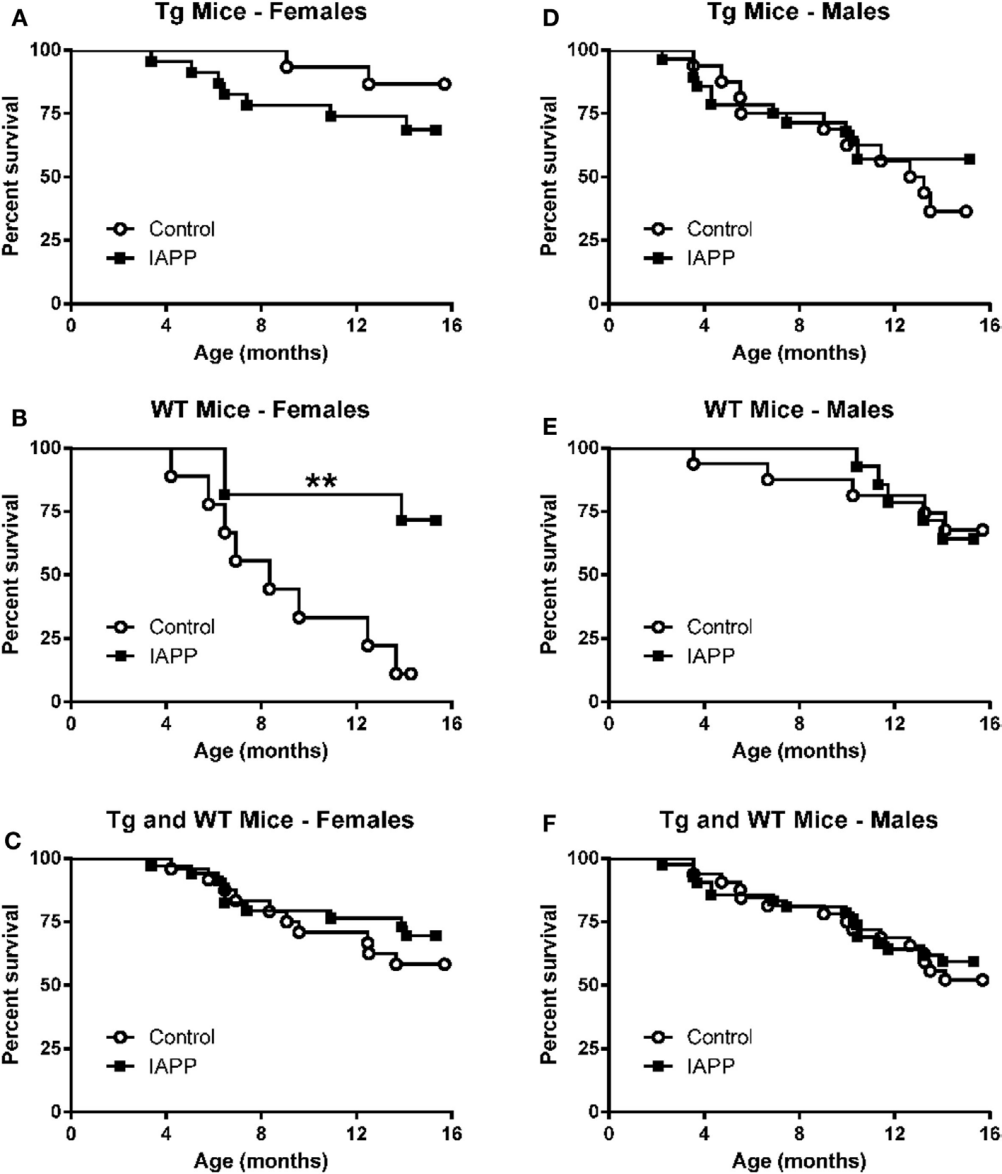
**Prophylactic IAPP vaccination promotes survival in WT female mice**. Kaplan–Meier survival plots for mice enrolled in the prophylactic IAPP study. **(A,C,D–F)** Tg female and male and WT male mice receiving IAPP treatment, as well as combined Tg and WT groups within each sex, had the same survival outcome as their control adjuvant-treated counterparts. **(B)** However, female WT IAPP-treated mice had a significantly better survival rate than WT control treated mice (*p* = 0.004).

Table 2 shows the numbers of mice of different sex and genetic background that were enrolled in the prophylactic IAPP vaccine study starting at 2–3 months of age. The experimental design was the same as in the IAPP_7-19-TT_ study. As in that study, a number of mice did not survive until the study endpoint (Table 2). Kaplan–Meier analyses of Tg and WT mouse deaths during prophylactic IAPP or control treatment was carried out (Figures 5A–F). Analysis of the survival curves prior to the glucose tolerance test or terminal sacrifice at 14 months of age indicated that a significantly greater proportion of WT female mice receiving the IAPP vaccine had survived till the study end point compared to WT female control-treated mice (*p* = 0.004, Figure 5B). As only one out of nine WT control females survived, a hazard ratio could not be calculated. Tg female and male mice and WT male mice receiving control or IAPP treatment, as well as combined Tg and WT groups within each sex, did not show any significant differences in their survival proportions during the course of the study (Figures 5A,C,D–F).

**Table 2:** **Mice enrolled and mouse deaths during the prophylactic IAPP study**.

Treatment	Enrolled	Female Tg	Male Tg	Female WT	Male WT	End of study euthanized	Female Tg	Male Tg	Female WT	Male WT	Deaths		
											Found dead prior to IPGTT	During/Post IPGTT	Other
Control	59	16	17	9	17	26	11	6	1	8	25	5	3
IAPP	78	24	28	12	14	44	14	14	7	9	27	5	2

As in the IAPP_7-19TT_ study, further analysis of the weight data showed a similar slope of weight gain in mice that died during the study compared to mice that survived until the end of the study (data not shown), suggesting that increased mortality could not be explained by abnormal weight gain and related complications or by weight loss because of disease in the colony.

### Weight – IAPP Study

To induce a diabetic phenotype, all the mice enrolled in the study were fed a high-fat diet from 2 months of age till the terminal endpoint. There were no significant differences in the weights of the groups (Figure S4 in Supplementary Material).

### Antibody Response and Plasma IAPP Levels – IAPP Study

**Figure 6:**
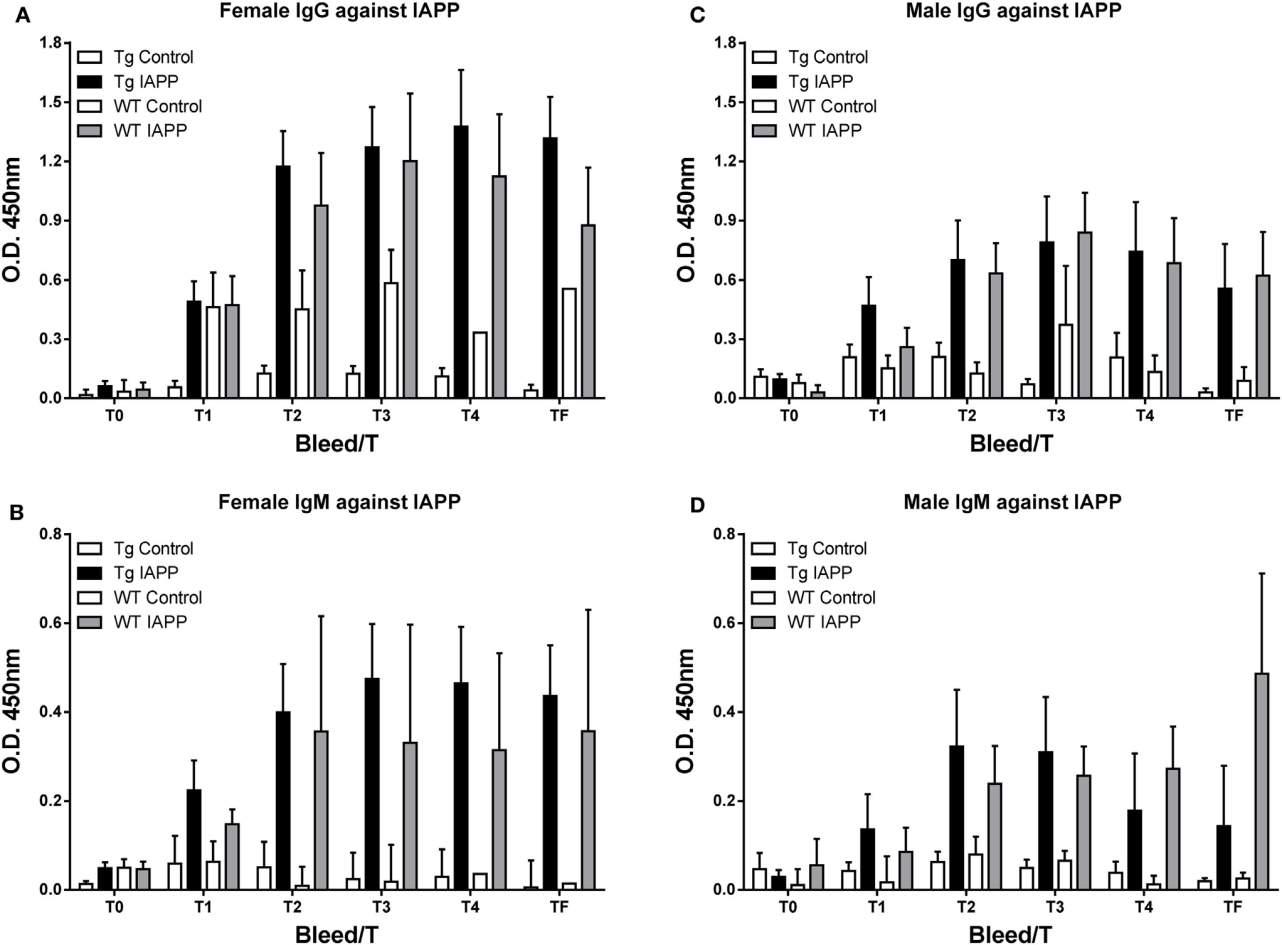
**The IAPP peptide is highly immunogenic in mice**. **(A–D)** Tg and WT mice were immunized from 2 months of age with alum adjuvant (Control) or IAPP peptide in alum adjuvant (IAPP). Mice were bled at regular intervals (T0–TF) and plasma samples (1:200) from the animals were analyzed by ELISA. IAPP-treated mice developed a strong and sustained immune response to the IAPP immunogen. **(A,C)** IgG levels were higher than **(B,D)** IgM levels. Error bars indicate SEM.

As expected, the antibody response toward the IAPP immunogen was stronger than toward this peptide in the IAPP_7-19-TT_ study as antibodies can be generated against the whole peptide (Figure 6). In the IAPP_7-19-TT_ study, a subpopulation of the antibodies is generated against the TT helper epitope resulting in a strong overall response against the immunogen, of which only some of the antibodies will recognize the 7-19 IAPP epitopes.

Random sampling of plasma samples from the study revealed increased plasma IAPP levels in immunized mice (WT: 514 (average) ± 214 (SEM) pmol/l, *n* = 7; Tg: 494 ± 370, *n* = 13) compared to control mice (WT: 12 ± 1, *n* = 4; Tg: 120 ± 92, *n* = 14), indicating target engagement of the antibodies generated toward the vaccine.

### Glucose Levels – IAPP Study

Measurements of plasma glucose levels during the course of treatment (12 months) showed that glucose levels decreased with time for female and male Tg mice (*p* < 0.0001) and male WT mice (*p* < 0.0001; Figure S5 in Supplementary Material). There were no differences in glucose levels over time in WT females (*p* = 0.26). Also, there were no significant differences between controls and IAPP immunized mice within each of these four groups. Neither was there an interaction between the two factors (bleeds versus treatment). Tfinal plasma glucose levels for IAPP-immunized mice were significantly greater than those in control mice for female Tg mice (*p* = 0.03; Figure S5). No treatment effect was observed on blood glucose levels from the terminal bleed in the other groups. This may suggest detrimental effects of the immunization in female Tg mice but as the mice are not developing a robust diabetic phenotype (rising blood glucose levels), it is difficult to interpret this finding. IAPP may have a role in glycemic regulation and could potentially prevent spikes in glucose levels after meals (3–5, 20). The immunization may enhance clearance of IAPP, which may result in increased glucose levels. Overall, as for the IAPP_7-19-TT_ study, based on these measurements, the mice are not developing a severe diabetic phenotype, in spite of being fed a high-fat diet and gaining weight during the course of the study. Hence, it may be difficult to assess treatment effect based on this parameter. The glucose tolerance test is more appropriate to detect subtle differences in diabetic phenotype.

### Glucose Tolerance Test – IAPP Study

One week prior to the end of the study period, mice underwent the IPGTT in the same manner as in the IAPP_7-19-TT_ study. Plasma glucose measurements were recorded prior to and at regular intervals up to 2 h post-delivery of the glucose bolus.

#### Females

**Figure 7:**
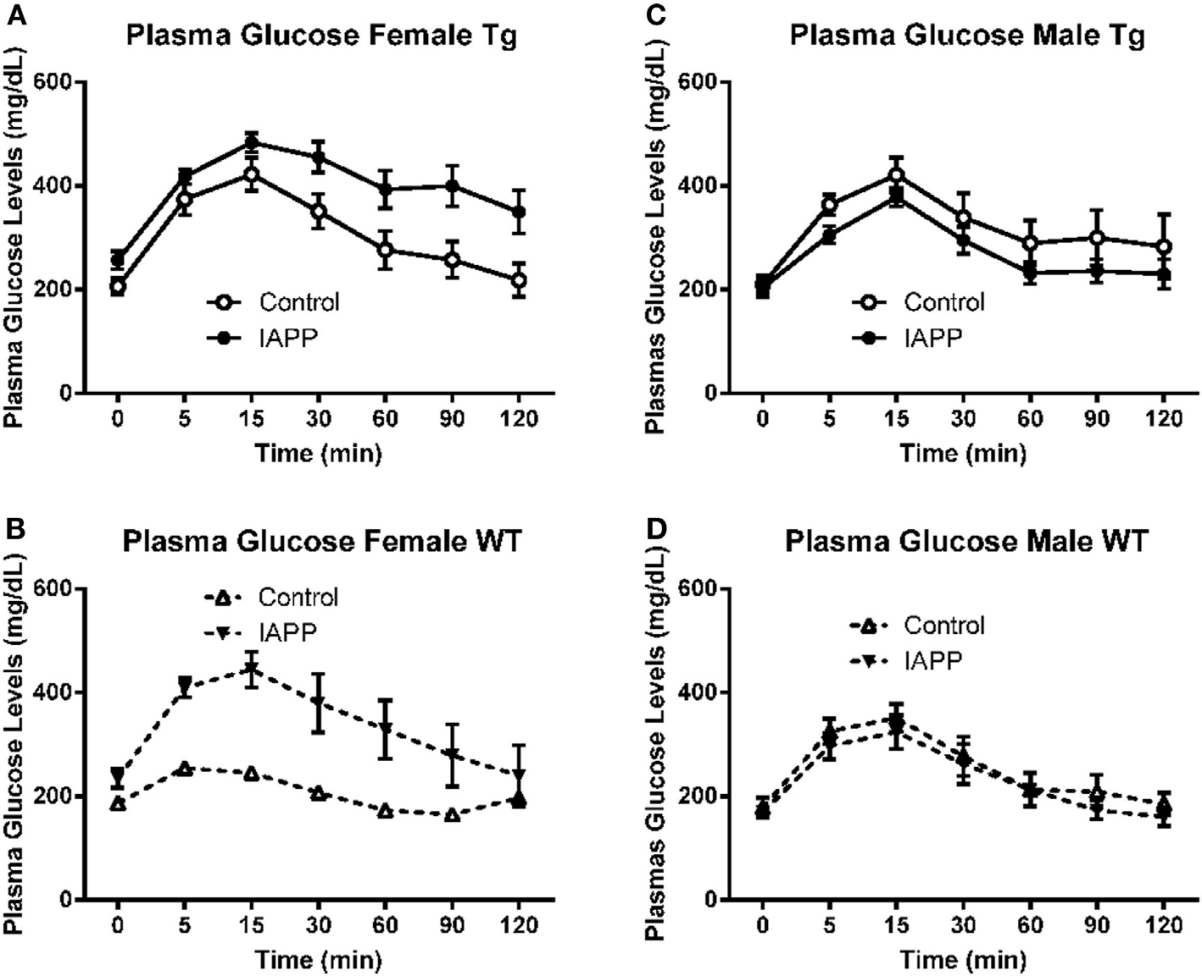
**Prophylactic IAPP treatment does not affect glucose clearance in Tg or WT mice**. At approximately 14 months of age, the diabetic status of the mice was assessed by the intraperitoneal glucose tolerance test (IPGTT). Mice were starved overnight and then bled to determine fasting plasma glucose levels (T0). They received a glucose bolus and were bled at various time points until T120 min. **(A–D)** IAPP immunized Tg females **(A)** were less able to regulate glucose levels than controls. The other groups **(B–D)** did not differ significantly. Error bars indicate SEM.

The test worked as expected as evidenced by the highly significant difference in glucose levels with time (*p* < 0.0001) in the Tg mice. IAPP-immunized Tg mice returned to basal plasma glucose levels significantly more slowly than control mice (treatment effect: *p* = 0.03; treatment × time interaction: *p* = 0.013, Figure 7A). This would suggest that IAPP immunized Tg females were less able to regulate glucose levels. Since only one female control WT mouse survived till the IPGTT test (Table 2; Figure 7B), statistical analysis of the results for WT female mice is excluded.

#### Males

Here too the test worked as expected as evidenced by the highly significant difference in glucose levels with time (*p* < 0.0001) in both the Tg and WT mice. In contrast to the females, Tg male IAPP immunized mice appeared to clear the glucose bolus more efficiently than Tg male controls. However, there was no significant treatment effect on plasma glucose levels (*p* = 0.153; Figure 7C). The WT immunized and controls did not differ significantly either (Figure 7D). Data were also analyzed as AUC but it did not provide further clarification.

### Insulin Levels – IAPP Study

Plasma insulin levels from the terminal bleeds were not significantly changed in the Tg or WT controls versus IAPP immunized mice (Figure S6 in Supplementary Material). Separating for sex, there were no significant differences either between these groups.

### Histology – IAPP Study

**Figure 8:**
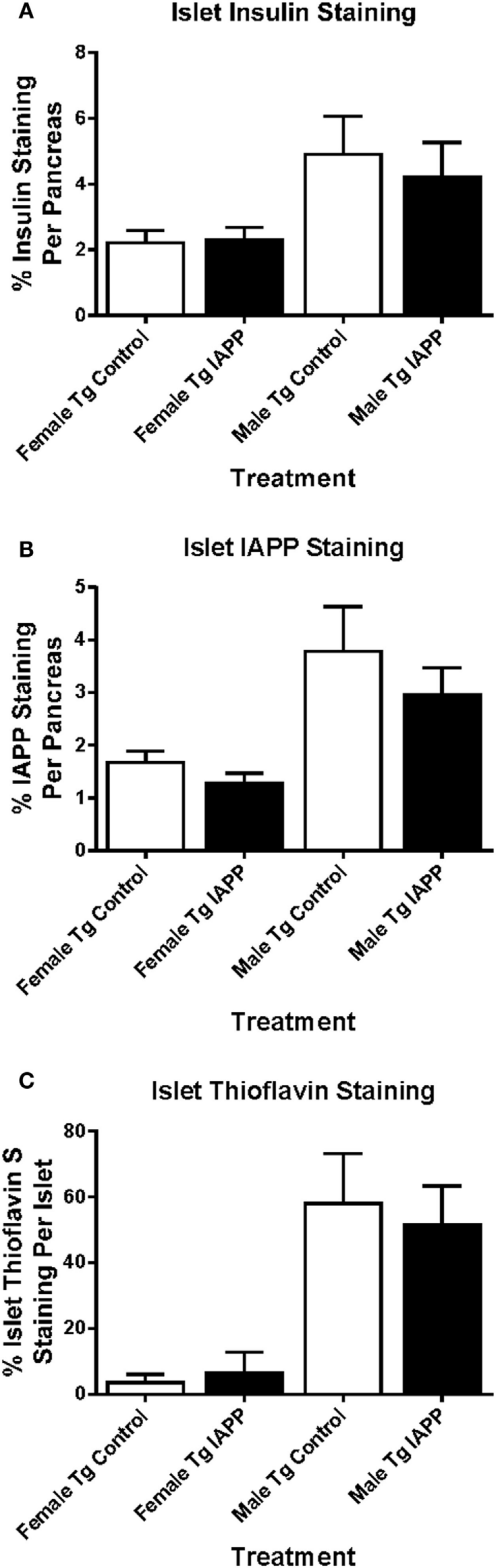
**Relative pancreatic β-cell area or amyloid burden are not changed in IAPP immunized Tg mice versus controls**. Pancreatic tissue was collected at the study endpoint and used for histological analysis of insulin, IAPP and amyloid load as determined by thioflavin S staining. **(A–C)** Relative islet β-cell area and amyloid burden were not altered in treated versus control mice. Error bars indicate SEM.

Insulin and IAPP immunostaining was performed on the Tg mice. There were no significant differences in the total area of insulin or IAPP staining between control and treated groups within each sex (Figures 8A,B).

As with islet area, there were no significant differences in amyloid burden between controls and IAPP immunized mice for either sex (Figure 8C). Insulin immunostaining of WT mice also showed no differences in islet area between control and treated groups within each sex (data not shown).

## Discussion

Islet amyloid polypeptide is co-secreted from pancreatic β-cells with insulin and converted to amyloid deposits in type 2 diabetes [reviewed in (21)]. The exact physiological function of IAPP is unknown, but it is thought to play a role in glucose homeostasis [reviewed in (22)]. The pathogenicity of islet amyloid aggregates has also been demonstrated. Insoluble IAPP fibrils were shown to be toxic to pancreatic islet cells (23), as were soluble oligomers (24), with both resulting in apoptotic β-cell death. Therefore, it has been suggested that islet amyloid may be an important link between insulin resistance and β-cell dysfunction in type 2 diabetes (25, 26). More recently, the focus has shifted to the role of IAPP oligomers in disease pathophysiology [reviewed in (7, 27)]. Thus, based on the current consensus, the aim of our study was to determine if immunotherapy targeting IAPP could be used as a novel treatment strategy to prevent fibrillogenesis of and/or remove IAPP aggregates and improve glucose regulation in type 2 diabetes.

Lin and colleagues had previously used a vaccination approach with “Alzheimer’s Aβ1-40 oligomers,” as the immunogen, to elicit the generation of anti-toxic oligomer antibodies and determine if IAPP deposits are formed intra- or extracellularly. Additionally, they hoped to prevent pancreatic β-cell loss (24). They showed that in two IAPP Tg mouse models, IAPP oligomers were detected intracellularly, and one of their models had extracellular IAPP amyloid deposits but vaccination with their immunogen had somewhat detrimental rather than protective effects (24). It is conceivable that their oligomeric immunogen may have had direct toxic effects on β-cells. Our approach was to design an immunogen that contains the B-cell epitope of IAPP but is void of the hydrophobic region that confers fibrillogenicity and thereby toxicity under certain conditions. This is analogous to our approach targeting Aβ in Alzheimer’s disease (10–12). To promote antibody response toward such a shortened sequence, tetanus toxin fragment (TT_947-967_) T-helper epitope that is commonly used in various marketed vaccines was attached to the B-cell epitope of IAPP.

The major benefit of our prophylactic IAPP_7-19-TT_ and IAPP vaccinations was prolonged lifespan of the WT female mice, importantly in two independent studies (*p* = 0.02 and *p* = 0.004), with the IAPP_7-19-TT_ immunization showing as well a strong trend (*p* = 0.056) for improved survival of Tg female mice. Indeed, the most significant effect on survival was observed when the Tg and WT female groups within that study were combined (*p* = 0.002). Hence, the surviving controls may not truly represent the group. It should be noted that all the mice were obese, having been on the high-fat diet throughout the study, and showed an increased rate of mortality compared to normal mice. To clarify why survival of WT female mice is extended with both vaccines, whereas apparently only with one of the vaccines in Tg females, will require additional studies in younger control mice to assess benefits prior to extensive mortality.

Due to the high mortality rates observed during the course of the study, we enrolled a total of 251 mice in the two studies (114 and 137, respectively), more than in any other report of this model. This large number of mice is likely more than in any hIAPP model report and most Tg mouse studies have much fewer mice. For example, Wang et al. used 15 hIAPP and 9 WT mice in their study (19), Hull et al. (15) used 48 hIAPP mice, and Zraika et al. (28) report on six mice per group. However, it would have been preferable to have had more mice in some of the groups for the endpoint measures.

The high mortality was seen both in Tg and WT mice and is, therefore, not related to the transgene. It can be explained by the long-term high-fat diet. Such high-fat diet induced mortality has been described in numerous studies. For example, Baur et al. (29) showed that resveratrol improved survival of mice on a high-fat diet. In that particular study, WT mice received high-fat diet from 50 weeks of age until 115 weeks of age with only about 40% of the mice surviving. We saw a similar percentage survival (about 30–50%) when the mice started on the diet at a young age (2 months) and stayed on it until 15–16 months of age. An example of other publications reporting similar reduced survival on a high-fat diet includes a study by Zhang et al. (30), who demonstrated that female WT mice fed a high-fat diet from 8 months of age had about 60% survival rate at 2 years, compared to control WT mice with about 90% survival rate. Another recent study from Baker et al. (31) described that WT mice on a 9% fat diet had a significantly reduced median lifespan (673 days) compared to mice on a 5% fat diet (897 days). An example from a disease model includes a study describing the effect of diet on TRAMP prostate cancer Tg mice fed from 4 to 50 weeks of age (32). In that particular report, control diet mice had a survival rate of 55%, but high-fat diet fed mice had only a 21% survival rate. There are other publications in which various Tg models have been fed a high-fat diet but for shorter periods of time, which precludes measuring survival rates. Examples of sex differences include a publication by Everitt et al. (33) showing high mortality in WT females (48%) receiving high-fat diet but not in males of a strain that is prone to develop myocardial disease, in females in particular. Additionally, in a non-mammalian model, Sun et al. (34) showed that a high-fat diet reduced the lifespan of female drosophila compared to males. While we saw reduced survival in both females and males on a high-fat diet, the effect was more pronounced in the females.

It is well established that high-fat diet promotes cardiovascular disease that may explain the enhanced mortality in the WT and Tg control groups. The beneficial effects of IAPP vaccination on survival are particularly strong in the females and need to be clarified in future studies.

We can rule out disease in the colony as a contributing factor as increased mortality was only seen in mice on high-fat diet and not in all groups and only some mice in each cage died. A similar slope of weight gain was observed in mice that died during the study, compared to mice that survived until the end of the study, indicating that increased mortality could not be explained by abnormal weight gain and related complications or by weight loss because of disease in the colony. The mice were housed in a pathogen-free facility, in which each cage has a separate air filtration built into the cage carts. Furthermore, controls and vaccinated mice were housed together, and the vaccination, in particular with the IAPP derivative, reduced mortality. Likewise, increased mortality cannot be explained by the adjuvant as both controls and vaccinated mice received the same amount of it and at the same intervals. We have used the same alum adjuvant in multiple studies in various Alzheimer’s models mice without obvious side effects (12, 35, 36). This type of adjuvant is approved for human use because of its safety. Additionally, repeated i.p. injections did not appear to result in peritonitis. We have used the same repeated i.p. injection approach in other studies using various mouse models on a regular diet without increased mortality (36, 37).

The second most significant benefit in the prophylactic studies was improved glucose clearance in IAPP_7-19-TT_-immunized Tg females, which interestingly was not observed in Tg males or WT mice. By carrying out the IPGTT, we were able to observe more subtle differences in the diabetic status of the mice. The most obvious explanation for these sex and model differences is that the Tg females appear to have more of a diabetic phenotype based on higher peak glucose values in the IPGTT assay, compared to identical males or WT mice.

Opposite effects on glucose clearance were observed in females receiving the IAPP vaccine. The beneficial effect of the immunization on glucose regulation in the Tg females in the IAPP_7-19-TT_ study and detrimental effects on the females in the IAPP study may be related to differences in antibody response. In the IAPP_7-19-TT_ group, modest antibody response was generated against IAPP that may be sufficient to clear soluble toxic IAPP aggregates while having minimal effect on normal IAPP that is needed for glycemic regulation [(38), and reviewed in (20)]. By contrast, in the IAPP group, moderate-to-high antibody response was generated against IAPP, which may reduce normal IAPP levels too much and thereby have adverse effects on glucose regulation. In support of this scenario, IAPP-deficient mice have been shown to develop a more severe form of alloxan-induced diabetes (39). This important finding suggests that in future clinical trials, care should be taken not to induce a robust immune response against IAPP that may have adverse effects on glucose regulation by interfering with the physiological role of IAPP. As this may prove difficult in individual subjects, passive immunization with IAPP targeting antibodies may be preferable.

In contrast to the females, the IPGTT for male mice in the studies did not indicate any differences between the two vaccines, IAPP_7-19-TT_ or IAPP, and their controls. It is not entirely clear why the immunotherapy was beneficial in the females and not in the males. One possibility is that it may be indirectly related to less amyloid burden in the females. The development of pancreatic IAPP amyloid deposits may actually be a way to sequester and contain in a relatively inert form more toxic smaller IAPP aggregates. As the females have less amyloid burden, they may have more of these smaller IAPP aggregates that may induce β-cell impairment and a diabetic phenotype as reflected in higher glucose levels in the females following a glucose bolus. The antibodies generated in response to the vaccination are likely to have better access to these smaller aggregates than the large amyloid deposits, and may, therefore, show beneficial effects in the females and not in the males.

In spite of the antibody response in the immunized mice and target engagement as evidenced by the related increase in plasma IAPP, several parameters were not affected by the immunizations, which may be explained by the relatively modest diabetic phenotype of this model. All the mice continued to gain weight as expected, since they were fed a high-fat diet. However, glucose levels were variable. In both studies, glucose levels decreased significantly over the time course of the study both in Tg and WT mice. This finding has also been reported by others in a large study of female C57BL/J6 mice fed a high-fat diet, in which circulating glucose levels declined throughout the 12-month study, although insulin levels did progressively increase (40). The reduction in glucose levels we observed may suggest that the mice are not developing a robust diabetic phenotype. It was, therefore, not surprising that the immunotherapy did generally not affect baseline glucose levels. An exception to this was observed in Tg females immunized with IAPP that had significantly higher resting glucose levels at sacrifice than identical controls. This increase was only observed at this time point and may relate to their impaired ability to clear a glucose bolus compared to their non-immunized controls.

Besides resting glucose levels, other markers of severe diabetes did not differ between treated and control mice, likely because this model does not have severe diabetes. Plasma insulin levels were only assessed from the final bleed at terminal sacrifice and were not altered in any of the mice tested. Relative β-cell area in the Tg mice, determined by staining for insulin and IAPP, showed no changes in female or male Tg mice receiving the prophylactic IAPP_7-19-TT_ or IAPP treatment, compared to controls. This was also the case with amyloid load. Overall, our findings indicate beneficial effects of IAPP-targeted immunization. The benefits depend on Tg status, sex, and immunogen. Considering these differences, it is advisable in future studies to pay close attention to these variables that clearly affect the therapeutic outcome. Our results are in contrast with a prior study that showed detrimental effects of immunization with a different immunogen in different models of type 2 diabetes (24). In conclusion, IAPP targeting immunotherapy may have benefits in humans suffering from type 2 diabetes. Further studies are warranted to investigate in more detail the mechanism by which such immunotherapy can promote survival, and improve glucose clearance.

## Author Contributions

PK performed various experiments, analyzed the data, and wrote the manuscript. HR, VG, WR, NA, and SK performed various experiments. HR, VG, NA, and SK analyzed the data. ES designed the immunogen, developed the original experimental design with input from other authors during the course of the studies, analyzed the data, and wrote the manuscript.

## Conflict of Interest Statement

The authors declare that the research was conducted in the absence of any commercial or financial relationships that could be construed as a potential conflict of interest.
