# Supplementary material for: Sex and Immunogen-Specific Benefits of Immunotherapy Targeting Islet Amyloid Polypeptide in Transgenic and Wild-Type Mice
Source: Front Endocrinol (Lausanne). 2016 Jun 14;7:62. doi: 10.3389/fendo.2016.00062 (PMC4907063; doi:10.3389/fendo.2016.00062)
Supplement: Supplementary file 1 [file Data_Sheet_1.docx]

Supplementary Material

Sex and Immunogen Specific Benefits of Immunotherapy Targeting Islet Amyloid Polypeptide in Transgenic and Wild-Type Mice

Pavan K. Krishnamurthy^1^, Hameetha B. Rajamohamedsait^1^, Veronica Gonzalez^1^, Wajitha J. Rajamohamedsait^1^, Nawal Ahmed^1^, Senthil K. Krishnaswamy^1^ and Einar M. Sigurdsson^1,2^*

*** Correspondence:** Professor Einar M. Sigurdsson: einar.sigurdsson@nyumc.org

## Supplementary Figures

**Supplementary Figure 2. Plasma Glucose Levels During Treatment Period - Prophylactic IAPP_7-19-TT_ Treatment**

**Supplementary Figure 3. TFinal Bleed Plasma Insulin - Prophylactic IAPP_7-19-TT_ Treatment**

**Supplementary Figure 4. Mouse Weights During Treatment Period - Prophylactic IAPP Treatment**

**Supplementary Figure 5. Plasma Glucose Levels During Treatment Period - Prophylactic IAPP Treatment**

**Supplementary Figure 6. TFinal Bleed Plasma Insulin - Prophylactic IAPP Treatment**

**
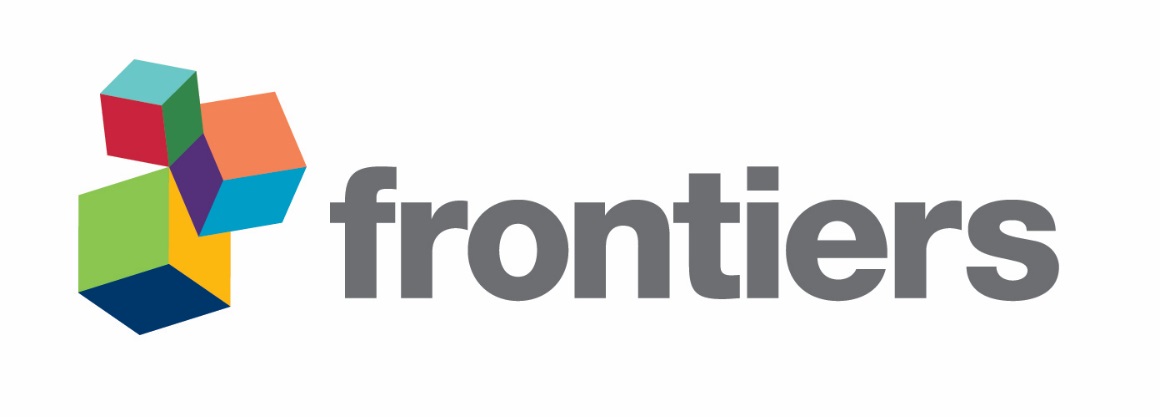
**

**Supplementary Figure 1:** Mouse weights during the course of the prophylactic IAPP_7-19-TT_ study. Mouse weights were measured every time the mice were bled during the course of the study. (A-D) There were no significant differences in weight between the control or IAPP_7-19-TT_ treated mice based on sex or genotype.

**Supplementary Figure 2:** Plasma glucose levels are reduced during the course of IAPP_7-19-TT_ treatment. (A-D) Tg and WT mice were immunized from 2 months of age with alum adjuvant (Control) or IAPP_7-19-TT_ peptide in alum adjuvant (IAPP_7-19-TT_). Mice were bled at regular intervals (T0 to T4) and their plasma glucose levels measured. Those trended to decrease in both control and IAPP_7-19-TT_ immunized mice of both sexes and backgrounds. (E) Plasma glucose levels were also measured from plasma samples obtained at the final bleed (Tfinal), and were not altered in control or IAPP_7-19-TT_ immunized mice of either gender or genotype. Error bars indicate SEM.

**Supplementary Figure 3:** Tfinal plasma insulin levels are not changed in control or IAPP_7-19-TT_ treated mice. There were no changes in plasma insulin levels at the terminal bleed between control or IAPP_7-19-TT_ treated mice (Tg or WT). Error bars indicate SEM.

**Supplementary Figure 4:** Mouse weights during the course of the prophylactic IAPP study. Mouse weights were measured every time the mice were bled during the course of the study. (A-D) There were no significant differences in weight between the control or IAPP treated mice based on sex or genotype.

**Supplementary Figure 5:** Plasma glucose levels are reduced during the course of IAPP treatment. (A-D) Tg hIAPP and WT mice were immunized from 2 months of age with alum adjuvant (Control) or IAPP peptide in alum adjuvant (IAPP). Mice were bled at regular intervals (T0 to T4) and their plasma glucose levels measured. Those decreased in control and IAPP immunized mice of either sex or genotype. (E) Plasma glucose levels were also measured at the final bleed (Tfinal). Those were significantly greater in only IAPP immunized female Tg mice (p = 0.03). Error bars indicate SEM.

**Supplementary Figure 6:** Tfinal plasma insulin levels are not changed in control or IAPP treated mice. There were no changes in plasma insulin levels at the terminal bleed between control or IAPP treated mice (Tg or WT). Error bars indicate SEM.
